# Supplementary material for: A Rapid Review of Interventions to Improve Care for People Who Are Medically Underserved with Multiple Sclerosis, Diabetic Retinopathy, and Lung Cancer
Source: Int J Environ Res Public Health. 2024 Apr 24;21(5):529. doi: 10.3390/ijerph21050529 (PMC11121396; doi:10.3390/ijerph21050529)
Supplement: Supplementary file 1 [file ijerph-21-00529-s001.zip › ijerph-2914905-supplementary.pdf]

Table S1. *Search concepts with MESH terms and keywords*

Search Concept	MESH Terms	Keywords
Multiple Sclerosis	"Multiple Sclerosis"[Mesh]	Multiple Sclerosis, multiple sclerosis†, Disseminated Sclerosis, MS, acute fulminating multiple sclerosis, relapsing-remitting multiple sclerosis, relapsing remitting multiple sclerosis, chronic progressive multiple sclerosis
Lung Cancer	"Lung Neoplasms"[Mesh]	lung neoplasm*, pulmonary neoplasm*, lung cancer*, pulmonary cancer*, cancer of the lung, cancers of the lung, cancer of lung, cancers of the lung
Diabetic Eye Disease	"Macular Edema"[Mesh] "Diabetic Retinopathy"[Mesh]	diabetic retinopath*, DR, diabetic macular edema,, DME,, irvine-gass macular edema, Irvine gass macular edema, pseudophakic macular edema,, cystoid macular edema, CME, macular edema,, cystoid macular dystrophy, cominant cystoid macular dystrophy, cystoid central retinal edema
Economic Factors	"Socioeconomic Factors"[Mesh] "Medically Uninsured"[Mesh]	socio-economic factor*, low-income population*, low income population*, indigen*, poverty, extreme poverty, absolute poverty, socioeconomic factor*, OR medical indigen*, standard of living, living standard*,
Uninsured		uninsured, medically underinsured, medically under-insured, under-insured, underinsured, un-insured
Lack of a medical home	"Patient-Centered Care"[Mesh]	medical home*, lack medical home, patient-centered care, patient centered care, patient-focused care, patient focused care
Rural Populations	"Rural Population"[Mesh] "Rural Health Services"[Mesh] "Rural Health"[Mesh]	rural population*, rural spatial distribution*, rural communit*, rural

	"Hospitals, Rural"[Mesh]	
People of color	"Health Disparity, Minority and Vulnerable Populations"[Mesh] "African Americans"[Mesh] "Blacks"[Mesh] "Indigenous Peoples"[Mesh] "American Indians or Alaska Natives"[Mesh]	African-American*, African American*, Afro-American*, Afro American*, People of color, person of color, persons of color, black people, black person*, indigenous people*, native American*, native people*, women of color, bipoc,
Continuum of Care	"Continuity of Patient Care"[Mesh],	care continuum, retention in care, care continuity,, patient care continuity, continuity of care, care retention, retention in care, insurance loss, loss of insurance, lack of insurance, coc, Continuum of Care
Diagnosis	Diagnosis"[Mesh] "Early Diagnosis"[Mesh] "Early Detection of Cancer"[Mesh]	early diagnosis, early detection of disease, disease early detection, "Health Surveys"[Mesh], health survey*, "Delayed Diagnosis"[Mesh], delayed diagnos*, late diagnos*, early screening<br>cancer early detection, cancer screening cancer screening test*, early diagnosis of cancer
Adherence and Compliance	"Treatment Adherence and Compliance"[Mesh] "Patient Compliance"[Mesh] "Patient Dropouts"[Mesh]	therapeutic adherence and compliance, treatment adherence, therapeutic adherence, , patient compliance, compliance, adherence, patient adherence, treatment compliance, therapeutic compliance, patient non compliance, patient non-compliance, patient noncompliance, non-adherent patient*, non adherent patient*, non-adherence, nonadherence, patient nonadherence, patient non-adherence, noncompliant patient*, non-compliant

		patient*, patient dropout*, retention in care, care retention, noncompliance
Disease Progression	"Disease Progression"[Mesh]	disease course, disease progression, disease progressions, progression, disease exacerbation, severity, disease severity
Implementation	"Implementation Science"[Mesh] "Technology Transfer"[Mesh]	Dissemination and implementation
Treatment follow-up	"Aftercare"[Mesh]	therapy, treatment, management, follow-up care, follow up care, loss to follow up, lost to follow up

* used in PubMed to denote truncation and search multiple variants of the word

† misspelling in the terms purposeful to capture possible errors in the articles and database terms

Table S2. Study Designs, Participants, Care Continuum, Level of Intervention, Interventions, Outcomes, and Findings

Author	Health condition	Design	Participants	Care continuum and level of intervention	Intervention type	Relevant outcomes	Main relevant findings
Baird, 2020 [21]	Multiple sclerosis	Quasi-experimental; single group, pre-post intervention	30 African American participants; 86.7% female, average age 44.3	Adherence to treatment; patient	Multicomponent intervention	Feasibility on four domains: process (recruitment and eligibility rate), resource (adherence, retention, time, cost), management (time, missing data), and scientific outcomes (safety, participant experience and burden, treatment effect). Scientific outcome—change in sedentary behavior.	The intervention was safe and feasible; small positive change in sedentary behavior.
Cascione, 2018 [22]	Multiple sclerosis	Randomized controlled trial (RCT); subgroup	141 African Americans; average age	Adherence to treatment; patient	Medication administration route	Adherence	At 48 weeks, higher adherence among those taking oral

		analysis of open-label	37.7 (fingolimod), 38 (iDMT); female 57% (fingolimod), 55% (iDMT)				versus injectable disease-modifying therapies (DMTs).
Hartung, 2020 [23]	Multiple sclerosis	Observational; retrospective cohort	39,661 Medicare beneficiaries; 91.9% White, 74.1% female, average age 66	Access to treatment; patient	Insurance subsidy	DMT initiation	Across demographics, newly diagnosed Medicare beneficiaries with reduced cost sharing via a low-income subsidy were more likely to initiate early self-administered DMT than those who did not have a low-income subsidy.
Kinnett-Hopkins, 2018 [24]	Multiple sclerosis	Observational; cohort study, single group pre-post intervention	32 Black participants; 94% female, average age 51.6	Adherence to treatment; patient	Multicomponent intervention	Feasibility: process (recruitment and retention), resources (communication and monetary costs), management (data management and safety reporting). Scientific outcomes—safety, burden, adherence, experience, and treatment effect.	The intervention was feasible, effective, and safe; exercise behaviors increased in inactive participants.
Plow, 2019 [25]	Multiple sclerosis	RCT; single blind, three parallel groups trial	208 participants; 90% White, 84% female, 23% rural, average age 52.1	Access to specialists; patient	Telemedicine	Fatigue impact, physical activity, and health-related quality of life	Fatigue management improved fatigue and quality of life at 12 weeks compared with social support, but not physical activity. Physical

							activity had improvement on quality of life compared with social support at 12 weeks.
Abramoff, 2018 [26]	Diabetic retinopathy	Observational; superiority study	900 patients; mean age 59; 47.5% male; 16.1% Hispanic, 28.6% African American	Diagnosis; system	Artificial intelligence (AI)-based interpretation of retinal images	Sensitivity and specificity	The AI system met the prespecified endpoints for superiority compared to the standard approach, with a sensitivity of 87.2% and specificity of 90.7%.
Alam, 2019 [27]	Diabetic retinopathy	Observational	115 images from 60 patients with diabetic retinopathy, 90 images from 48 patients with sickle-cell retinopathy, and 40 images from 20 control patients	Diagnosis; system	AI-based interpretation of optical coherence tomography angiography (OCTA) imaging	Sensitivity and specificity	The AI model has 97.84% sensitivity and 96.88% specificity for diagnosing disease versus normal control, 95.01% sensitivity and 92.25% specificity for correctly classifying diabetic versus sickle-cell retinopathy, 92.18% sensitivity and 91.6% specificity for staging diabetic retinopathy, and 93.19% sensitivity and 91.6% specificity for staging sickle-cell retinopathy.
Al-Aswad, 2021 [28]	Diabetic retinopathy	Pilot study	957 people; 31% African American,	Screening; system	Mobile clinic	Patient follow-up rates after diabetic retinopathy screening	6% of patients screened positive for diabetic retinopathy

			46% Hispanic; 54% female				and were referred for follow-up. Of these, 48% were reached for individual follow-up, and 71% of those reached completed ophthalmologist follow-up. Diabetic retinopathy was confirmed in 79% of those reached, and additional eye problems were detected in 43%.
Daskivich, 2017 [29]	Diabetic retinopathy	Quasi-experimental; pre- and post-intervention comparison	600 patients; 63% female; 74% Hispanic or Latino	Screening; organization	Teleophthalmology screening in primary care	Screening rates and change in wait time	16.3% increase in annual screening rates and 89.2% wait-time reduction.
Hatef, 2017 [30]	Diabetic retinopathy	Quasi-experimental; pre- and post-intervention comparison	Total 441 patients: 213 patients in 2010, mean age 51.4, 82.6% female; 228 patients in 2012, mean age 52.3, 82% female	Screening; organization	Teleophthalmology screening in primary care	Efficacy of program, defined as the number of patients with completed annual exams divided by number of patients with diabetes seen	Annual exam completion rate increased from 47.9% to 78.1% ($p < 0.001$; *OR* 4.98); 12% of those with diabetic retinopathy identified in their scan and were referred to ophthalmologists completed the referral.
Jani, 2017 [31]	Diabetic retinopathy	Quasi-experimental; pre- and post-intervention comparison	1,787 patients; mean age 55.4; 62.7% female;	Screening; organization	Teleophthalmology screening in primary care	Screening rates among eligible patients; factors associated with need	Pre-implementation screening rate of 25.6%, postimplementation screening rate of

			55.4% African American; 72.8% publicly insured or uninsured; 64.5% racial/ethnic minorities			for referral to ophthalmologist	40.4%; 11% of screened patients had diabetic retinopathy without referral; 9.3% had diabetic retinopathy with referral. Of those referred, 60% completed referral visit within study period.
Liu, 2019 [32]	Diabetic retinopathy	Qualitative; patient and provider interviews	Patients (*n* = 20): median age 67, 55% male, 100% White, non-Hispanic; primary care provider (PCP) (*n* = 9): 77.8% male	Screening; organization	Teleophthalmology screening in primary care	Barriers and facilitators to use of teleophthalmology	Patient barriers included unfamiliarity with teleophthalmology, misconceptions about screening, and logistical difficulties. Facilitators included primary care provider (PCP) recommendation and convenience factors. PCP barriers included not knowing when screening was due and unfamiliarity with teleophthalmology. Facilitators included ease of referral process and communication of results.
Mehranbod, 2019[33]	Diabetic retinopathy	Randomized controlled trial (RCT)	301 patients; among those randomized to	Screening; organization	Automated telephone appointment reminder	Appointment attendance rates	Among those receiving usual reminders, attendance rates for

			intervention, mean age 56.9, 32.4% African American, 64.7% Latino, 62.6% female; among those randomized to usual care, mean age 57.2, 27.7% African American, 69.2% Latino, 61.5% female				appointments were lower among African American patients compared with Latino patients. Adding automated reminders improved attendance and narrowed the disparity in rates between African American and Latino patients; rates for both groups remain low, at 51.6% and 62.3%, respectively.
Ramchandran, 2020 [34]	Diabetic retinopathy	Mixed-methods; survey and discussion	23 patients; mean age 56, 53% female, 57% African-American, 34% Medicaid	Screening; organization	Teleophthalmology screening in primary care	Perceptions and view of teleophthalmology	Patients rated teleophthalmology as highly as regular care, perceived high value of teleophthalmology, and were willing to pay an equivalent copay.
Rowe, 2021 [35]	Diabetic retinopathy	Observational; retrospective descriptive	875 patients; median age 49, 51.1% female, 31.9% African American, 21.5% Hispanic, 39.2% uninsured,	Screening; system	Mobile clinic	Relative value units and dollar value of service provided at the eye clinic	Relative value units of 1,271.3 for services from October 2013 to February 2020; dollar value equivalent of $119,263.16, or $136.30 per screening.

			12% Medicaid				
Serrano, 2018 [36]	Diabetic retinopathy	Observational; pre- and post-intervention surveys	247 patients; 59% female; 89% over age 50	Screening; organization	Teleophthalmology screening in primary care	Patient satisfaction	Patients expressed satisfaction with telemedicine and preferred it to in-person visits; patients who had prior face-to-face exams were less likely to prefer telemedicine.
Tan, 2021 [37]	Diabetic retinopathy	Observational; pilot study	118 patients; average age 45, 74% male, 36% Hispanic or Latino	Diagnosis; system	Mobile clinic	Agreement of optical coherence tomography (OCT) and eye exams for detecting retinal abnormalities	OCT and clinical exam likely complement each other to detect diabetic retinopathy. Among patients with diabetes, OCT and clinical exam had moderate agreement in diagnosing retinopathy, with a third of diagnoses detected by OCT alone, a third by clinical exam alone, and a third with both.
Bagcivan, 2018 [38]	Lung cancer	Qualitative analysis of randomized controlled trial (RCT) data	142 people: 51% female, 66% rural dwelling, average age 68	Access to specialists; system	Access to palliative care specialists	Evaluation and treatment recommendations made in early outpatient palliative care consultations	Early consultations addressed patient and family concerns not typically addressed in cancer care visits. Commonly evaluated symptoms were mood, general pain, and

							cognitive/mental status.
Beer, 2020 [39]	Lung cancer	Qualitative; focus groups	11 lung cancer survivors: 64% Black or African American, 73% female, average age 64.7;<br>8 family members: 75% Black or African American, 63% male, average age 58.6	Access to treatment; patient	mHealth app	Perceived barriers and facilitators of acceptance of the app	Primary benefits were convenience and having credible health information; top concerns included cost and difficulty of use.
Cardarelli, 2020 [40]	Lung cancer	Observational; retrospective cohort	774 people with lung cancer: 91.2% White, 50% male, average age 63	Screening; organization	Screening methods	Subsequent testing and cancer diagnosis	Use of Lung-RADS led to 13.3% fewer additional tests compared with NLST. Among those with additional testing, the number identified with cancer was five times higher using Lung-RADS compared to NLST.
Cykert, 2019, 2020 [41,42]	Lung cancer	Longitudinal pragmatic trial	2019—360 people with breast or lung cancer with the intervention: 31.7% Black, 44.4% female,	Access to treatment; system	Multicomponent quality improvement-based intervention	2019—treatment completion; 2020—receipt of curative treatment	2019—Among patients in the intervention group, treatment completion rates did not differ between Black and White patients (*OR* = 0.98;

			average age 66.2; 2,841 people with breast or lung cancer as historical controls: 15.9% Black, 50.4% female, average age 68.8; 597 people with breast or lung cancer as concurrent controls: 13.2% Black, 46.7% female, average age 69.5. 2020—360 patients in intervention: 32% Black; 2,841 patients in the retrospective group: 16% Black				95% CI: 0.46, 1.2). Among patients in the historical and concurrent control groups, Black patients had reduced treatment completion compared with White patients (*OR* = 0.79; 95% CI: 0.65, 0.96; *OR* = 0.69; 95% CI: 0.49, 096).<br><br>2020—Black and White patients in the intervention group had similar receipt of curative treatments. Black patients in the retrospective group had lower rates of receiving curative treatment compared with White patients (*OR* = 0.66; 95% CI 0.51, 0.85).
Erkmen, 2021 [43]	Lung cancer	Quasi-experimental; feasibility study	505 people: 56.8% African American, 51% women, median age of 45	Screening; organization	Community engaged LDCT screening program	Adherence to follow-up care, cancer diagnosis, cancer treatment, and smoking cessation	At 1 year, all people with Lung-RADS categories 3 or 4, but only 23.7% of those with negative screens, adhered to follow-up screening. At 2 years, only 35.4% with positive

							screens and no cancer followed up.
Fung, 2018 [44]	Lung cancer	RCT	400 Chinese Americans under age 65	Screening; patient	Patient education	Changes in knowledge, attitudes, and screening completion/intent	Seminars developed for Cantonese-speaking Chinese Americans had an impact on the beliefs and stated behaviors of Chinese Americans, although at baseline more than two thirds were aware of availability of screening tests. Both groups had high knowledge at baseline. Changes in knowledge, attitudes, and screening intent were minimal between groups.
Huang, 2021 [45]	Lung cancer	Observational; case control	956 people: 51% female, 39% Appalachian dwelling	Access to specialists; organization	Molecular tumor board	Survival	Compared to those with reviews, those without reviews had poorer survival (HR = 8.61; 95% CI: 3.83, 19.31).
Lau, 2021 [46]	Lung cancer	Quasi-experimental; pre- and post-comparisons	74 African American patients: 51% male	Screening; patient	Decision aid	Knowledge, decisional conflict, and acceptability	Use of the decision aid led to small improvements in knowledge and increased concordance with current recommendations. Knowledge about risk factors and screening improved 25% after viewing

							the decision aid, decisional conflict decreased by 49%, concordance between individual preference and eligibility for screening increased from 21% to 33%, and 93% of participants said the tool helped them consider screening.
Li, 2020 [47]	Lung cancer	Mixed-methods; surveys and focus groups	50 Chinese Americans, 84% male, average age 70.4	Screening; patient	Decision aid	Knowledge and beliefs associated with lung cancer; perceived benefits of lung cancer screening; barriers and facilitators to lung cancer screening; evaluation of the translated and adapted AHRQ decision aid; elements of a successful lung cancer screening program	Participants reported that the adapted tool would facilitate informed decision making for LDCT screening. Based on reviewing the decision aid, more than 80% understood causes and symptoms of lung cancer and LDCT screening and associated benefits, harms, and insurance coverage, although 66.7% were unable to understand the content without help.
Liu, 2020 [48]	Lung cancer	Observational; retrospective data analysis	101,227 patients aged 20–64 diagnosed with non-	Diagnosis; system	Medicaid expansion	Early-stage diagnosis and 2-year survival	Compared to men in states that didn't expand Medicaid, those in states that did expand Medicaid had greater increases

			small-cell lung cancer				in 2-year survival and early-stage diagnosis. Outcomes for women did not differ among states that did and did not expand Medicaid.
Loehrer, 2018 [49]	Lung cancer	Quasi-experimental	67,987 patients: average age 54.8	Access to treatment; system	Insurance expansion	Utilization of high-volume hospitals, rates of complex cancer operations, and disparities in use of high-volume hospitals by insurance	Rates of complex surgical care increased by 14% relative to non-expansion states (IRR = 1.14; 95% CI 1.03, 1.27). The probability of undergoing surgical resection at high-volume hospitals did not change.
Olazagasti, 2021 [50]	Lung cancer	Observational; retrospective review	530 patients with lung cancer	Screening; organization	Screening methods	Screening eligibility	Among patients already diagnosed with lung cancer, significantly more Hispanic/Latinx patients did not qualify for screening based on USPSTF guidelines compared with patients of other races. Rates of eligibility did not differ between African Americans and those who were White, Asian, or other races comparing the NCCN or USPSTF guidelines.

Otto, 2021 [51]	Lung cancer	RCT; secondary data analysis	137 Black patients and 21 non-Black oncologists	Adherence to treatment; patient	Presence of a companion	Communication behavior, time spent with patient, patient confidence in recommended treatment, oncologist perceptions of patient adherence and social support	When a companion was present, oncologists provided more patient-centered communication and spent more time with patients. They perceived patients to be more active participants and to have more social support.
Owens, 2020 [52]	Lung cancer	Qualitative study	Six lung cancer survivors and six family members	Access to treatment; patient	mHealth app	Cultural sensitivity, quality of life, acceptability	The majority of participants thought the app was appropriate for African Americans, the information was well understood, and that it would benefit lung cancer survivors to use the app. Participants were receptive to using the app but raised concerns of health literacy for others.
Pasquinelli, 2020 [53]	Lung cancer	Observational; retrospective cohort	883 people: 56.3% African American, 55.8% men, average age 64.8	Screening; organization	Screening methods	Sensitivity	Among African American patients, the PLCO model had higher sensitivity for lung cancer screening compared with the UPSTSF guidelines ($p < 0.0001$).
Percac-Lima, 2018 [54]	Lung cancer	RCT	1,200 current smokers. 400	Screening; organization	Patient navigator	Proportion of patients receiving screening	The proportion of patients receiving

			people in intervention: 77.8% White, 47% female, average age 61.8; 800 people in control group: 83.3% White, 55.3% female, average age 62.4			via computed tomography (CT) scan	CT screening via chest or lung CT was higher among those receiving the patient navigator compared with those receiving usual care ($p < 0.001$).
Prosper, 2021 [55]	Lung cancer	RCT; secondary data analysis	Among the 53,452 National Lung Screening Trial participants, 2,376 Black, 54.7% male, average age of 60.5	Screening; system	Screening methods	Population-based changes in lung cancer mortality	Compared with the original NLST results, among the synthesized population that mirrored the U.S. population of 13.4% Black individuals, LDCT screening had a greater relative reduction in lung cancer mortality (HR = 0.82; 95% CI 0.72, 0.92 vs. full NLST cohort HR = 0.84; 95% CI 0.76, 0.96).
Raghavan, 2020 [56]	Lung cancer	Observational	550 people: 20% Black, 66% Medicaid, 34% uninsured, 70% rural	Screening; system	Mobile LDCT screening	Lung cancer screening results	Screening identified 601 pulmonary nodules, including 267 participants with Lung-RADS 1, 183 participants with Lung-RADS 2, 62 participants with Lung-RADS 3, and 38 participants with

							Lung-RADS 4 lesions. Among those screened, 12 had lung cancer. Primary non-small-cell lung cancers were found in 6 people, an additional 5 people had metastatic non-small-cell lung cancers, and 1 person had metastatic small cell undifferentiated lung cancer.
Randhawa, 2018 [57]	Lung cancer	Quasi-experimental; feasibility study	169 patients: 65.1% Black, 5.3% Hispanic, 3.6% Asian, 62.1% female, average age 63.99	Screening; organization	LDCT screening	Descriptive statistics of screened patients, referrals to screening program, and perceived barriers to lung cancer screening	Screening identified 18.3%, 68.6%, 9.5%, and 3.6% of participants as Lung-RADS 1, 2, 3, and 4, respectively. Among physicians surveyed (*n* = 19; 22% response rate), 15% had never referred a patient. Barriers to referral included time constraints and precertification requirements.
Sender, 2019 [58]	Lung cancer	Quasi-experimental	544 rural patients ages 55–77	Screening; organization	QI feedback to providers	Screening rates	After 6 months, screening rates improved to 85%, compared with 68% prior to the intervention.
Sferra, 2021 [59]	Lung cancer	RCT	237 people: 61.6% African	Screening; patient	Decision aid	Decision regret and knowledge of screening	Patients randomized to Option Grids had lower decision regret

			American, 54% male, average age 64				and higher knowledge regarding next steps for positive screens and potential need for invasive procedures compared with those randomized to ShouldIScreen ($p = 0.0198$ and $p = 0.02$).
Springer, 2018 [60]	Lung cancer	Observational	White, ages 55–80	Screening; system	Awareness campaign	Awareness of lung cancer screening	Screening rates could not be calculated due to data suppression rules. Evidence did not show differences in screening rates between patients with more than and less than a 30-year smoking history.
Thurlapati, 2021 [61]	Lung cancer	Observational; retrospective cohort	980 patients	Screening; organization	Screening methods	Eligibility for screening among patients with known lung cancer	One third of patients diagnosed with lung cancer did not meet the 2103 screening guidelines. Using the revised NCCN guidelines, 12.5% who were ineligible for screening would have been qualified for LDCT and potentially detected cancer at a younger age and lower stage; however, 87.5% of those patients with lung cancer who

							were missed would still not have met screening criteria. Among those who did not meet screening guidelines, 50% were African American, 95% had a smoking history, and 80% were diagnosed with advanced stage lung cancer.
Townsend, 2021 [62]	Lung cancer	Observational; retrospective analysis	7,807 patients: 99% White, 60% male, average age 63	Screening; organization	LDCT screening	Number of patients screened, screens with follow-up testing, lung cancer detections	In 8 years, 7,807 screens completed, with 1.55% having a diagnostic or therapeutic procedure after LDCT. Lung cancer was detected in 1.4% of screens.
Williams, Looney, 2021 [63]	Lung cancer	Quasi-experimental; pre-post surveys	481 patients; 93% African American, 73.1% female, average age 58.3	Screening; patient	Educational sessions	Knowledge of lung cancer screening guidelines	Among participants, there was an increase in knowledge, perceived benefits of lung cancer screening, and self-efficacy as well as decreased perceived barriers ($p = 0.001$).
Williams, Shelton 2021 [64]	Lung cancer	Quasi-experimental; pre-post surveys	77 people: 46% White, 22% Black/African American, 22% Latino, 68% female,	Screening; patient	Educational sessions	Knowledge of lung cancer screening guidelines and stigmas of cancers, reach, efficacy, adoption, and implementation	Community health workers and partnering with local health systems and communities helped reach more patients and educated them

			average age 44.8				on cancer screenings. Participants improved on some, but not all, knowledge, benefit, and stigma measures.

Lung-RADS = Lung Imaging Reporting and Data Systems; NLST = National Lung Screening Trial; RCT = randomized controlled trial; LDCT = low-dose computed tomography; AHRQ = Agency for Healthcare Research and Quality; USPSTF = United States Preventive Services Task Force; NCCN = National Comprehensive Care Network
